# First Animal Source Metagenome Assembly of *Lawsonella clevelandensis* from Canine External Otitis

**DOI:** 10.3390/pathogens14050465

**Published:** 2025-05-10

**Authors:** Adrienn Gréta Tóth, Norbert Solymosi, Miklós Tenk, Zsófia Káldy, Tibor Németh

**Affiliations:** 1 Centre for Bioinformatics, University of Veterinary Medicine, 1078 Budapest, Hungary; solymosi.norbert@gmail.com; 2 Department of Physics of Complex Systems, Eötvös Loránd University, 1117 Budapest, Hungary; 3 Department of Microbiology and Infectious Diseases, University of Veterinary Medicine, 1143 Budapest, Hungary; tenk.miklos@univet.hu; 4 Department and Clinic of Surgery and Ophthalmology, University of Veterinary Medicine, 1078 Budapest, Hungary; zsofi.kaldy@gmail.com (Z.K.); nemeth.tibor@univet.hu (T.N.)

**Keywords:** *Lawsonella clevelandensis*, otitis externa, dog, nanopore sequencing, metagenome-assembled genome

## Abstract

External otitis is one of the most common conditions in dogs to be presented to the veterinarian. Moreover, the disorder is often challenging to manage. The range and role of microorganisms involved in the pathogenesis are currently not fully understood. Therefore, the condition has been studied using third-generation sequencing (Oxford Nanopore Technology) to gain a more complete picture of the pathogens involved. Throughout the metagenome assembly of a sample from the ear canal of an 11-year-old female Yorkshire terrier suffering from chronic external otitis, a genome of *Lawsonella clevelandensis* was compiled. To our knowledge, this result is the first of its type of animal origin. The outcome of the assembly is a single circular chromosome with a length of 1,909,339 bp and 1727 predicted genes. One open reading frame associated with antimicrobial resistance could have been identified. Comparing all available genomes, the species can be associated with three main genome clusters. The finding contributes to the extending knowledge bank about this often-overlooked pathogen and raises attention to the role of nanopore sequencing by the identification and characterization of microorganisms that are difficult to culture.

## 1. Introduction

External otitis is a common condition in dogs. The conventional treatment is occasionally rather challenging and often fails. This is partly due to the anatomical characteristics of the ear and partly due to the difficulty in identifying the microorganisms (bacteria, fungi) involved in the development of the disease. Additionally, there is a clear link between widely occurring food allergies and the prevalence of otitis externa in dogs [1]. However, other sources of dermal hypersensitivity may also play a role in the clinical scenario. In the case of purely infective external otitis, the lack of information about the drug susceptibility of the pathogens involved in the disease is an additional therapeutic challenge. The above-mentioned factors can lead to so-called end-stage chronic otitis requiring an invasive salvage procedure, the Total Ear Canal Ablation (TECA) with or without Lateral Bulla Osteotomy (LBO) [2].

Classic microbiological tests (e.g., culturing, polymerase chain reaction (PCR)) can be used as a model-based approach to identify the microorganisms located both in the external ear canal and the tympanic bulla. However, the bacteria involved have different growth characteristics and require different culture conditions, or cannot be cultured at all using classical techniques. Furthermore, the commensal bacteriota may mask the real causative agent or other microbial factors in multi-microbial origin cases. At the same time, next-generation and third-generation sequencing allow a data-driven approach rather than a model-based approach, providing a wider view of the microbiota under study. Digital data generated by partial or complete sequencing of nucleic acids (DNA and/or RNA) extracted from a sample can be compared with known genomes to build up a more exact and real-time cross-sectional microbiological image. In clinical metagenomics [3], the genomic sequencing of microorganisms provides information on their genetic characteristics (e.g., virulence and antimicrobial genes) in addition to their presence and abundance. Moreover, for metagenomic studies, Oxford Nanopore Technology (ONT) takes less time than the conventional microbiological methods [4]. The data presented here are from a larger series of clinical metagenomic samples from canine otitis externa cases. The sample set is collected to investigate the pathogens involved in the condition. From one of the external auditory canal samples, an essentially complete genome of the emerging pathogenic bacteria *Lawsonella clevelandensis* [5] could have been generated by metagenome assembly. *L. clevelandensis* is a Gram-positive, anaerobic bacterium that was first described in 2016 after being isolated from human abscesses [5]. It belongs to the family Corynebacteriaceae and exhibits features intermediate between typical actinomycetes and mycobacteria. The bacterium is slow-growing and often overlooked in routine cultures, which can complicate diagnosis [5]. Although its natural habitat is not fully known, *L. clevelandensis* is believed to be part of the human microbiota, potentially residing on the skin or mucous membranes [5,6,7]. The aim of this study was to characterize an accidental finding; *L. clevelandensis* is a less-studied organism in animal diseases and the goal was to position the identified animal-derived strain among its human-derived relatives. Furthermore, the study raises attention to the potential of rapid microbial diagnostics for difficult-to-culture microorganisms.

## 2. Materials and Methods

### 2.1. Sample Collection

The metagenomic sample was collected from the right external ear canal of an 11-year-old intact female Yorkshire terrier before TECA/LBO surgery. The dog was previously diagnosed with ear canal stenosis due to end-stage external otitis. The duration of the signs was more than a year. No tumor tissues were identified via histopathology examination. A presumptive diagnosis was chicken meat allergy, and multiple conservative treatment efforts had been performed previously. The skin of the ear canal was red, swollen, scaled, thickened, and covered with purulent ear discharge before the surgery. By the routine bacteriology testing, *Pasteurella multocida* and mixed anaerobic bacteriota were detected from the sample.

### 2.2. DNA Extraction, Library Preparation, and Sequencing

DNA extraction was performed with the QIAamp PowerFecal Pro DNA Kit (Qiagen, Hilden, Germany) according to the manufacturer’s instructions. The concentrations of the extracted DNA solutions were evaluated with an Invitrogen Qubit 4 Fluorometer (Thermo Fisher Scientific, Waltham, MA, USA) using the Qubit dsDNA HS (High Sensitivity) Assay Kit (Thermo Fisher Scientific, Waltham, MA, USA). The metagenomic long-read library was prepared by the Rapid Barcoding Kit 24 V14 (SQK-RBK114.24) (Oxford Nanopore Technologies (ONT), London, UK). The sequencing was implemented with a MinION Mk1C sequencer (Oxford Nanopore Technologies, London, UK) using an R10.4.1 flow cell from ONT.

### 2.3. Bioinformatic Analysis

The basecalling was performed using dorado (https://github.com/nanoporetech/dorado (accessed on 1 December 2023), v0.4.3) with model dna_r10.4.1_e8.2_400bps_fast@v4.2.0, based on the POD5 files converted from the FAST5 files generated by the sequencer. The raw reads were adapter-trimmed and quality-based filtered by Porechop (v0.2.4, https://github.com/rrwick/Porechop (accessed on 2 January 2024)) and Nanofilt (v2.6.0, minimal Q = 7, length = 50) [8], respectively. To obtain an overview of the bacterial composition, the cleaned reads were taxonomically classified by Kraken2 [9] with the National Center for Biotechnology Information (NCBI) non-redundant nucleotide database [10]. The assembly of the cleaned reads was carried out using Flye [11] (v2.9-b1779). The polishing of the contigs was performed with Racon (https://github.com/isovic/racon (accessed on 2 January 2024), v1.4.3) and medaka (https://github.com/nanoporetech/medaka (accessed on 2 January 2024), v1.7.2). Contigs were indexed, mapped, and sorted using minimap2 v2.26 [12] and SAMtools v1.19 [13] for downstream binning. The metagenomic binning was performed on the contigs using seven different tools: MetaBAT2 [14], MaxBin2 [15], CONCOCT [16], MetaBinner [17], MetaDecoder [18], LRBinner [19], and SemiBin2 [20]. All bins were integrated using DAS Tool [21]. Taxonomic assignment was performed using GTDB-Tk v2.1.0 [22]. To assess taxonomic consistency and metagenome-assembled genome (MAG) quality, high-quality bins were further validated using CheckM2 [23].

The average nucleotide identity (ANI) of the *L. clevelandensis* classified bin compared to the genome was estimated by pyani (v0.2.12) [24]. The contigs were subjected to virulence gene and antimicrobial resistance gene (ARG) analysis. Virulence genes were screened using Abricate (v1.0.1, https://github.com/tseemann/abricate, accessed on 14 April 2025) with the Virulence Factor Database (VFDB, downloaded on 27 November 2024) [25]. All possible open reading frames (ORFs) were predicted by Prodigal (v2.6.3) [26] on each contig. The protein-translated ORFs were searched for ARG sequences against the Comprehensive Antibiotic Resistance Database (CARD, v.3.2.8) [27,28] by the Resistance Gene Identifier (RGI, v6.0.3). Plasmids were screened using Plasflow (v1.1) [29] and the Basic Local Alignment Search Tool (BLAST) [30] for nucleotide-to-nucleotide comparisons, and phages were screened using Virsorter (v2.2.4) [31] and CheckV (v1.0.1) [32]. For the genome annotation, Prokaryotic Genome Annotation Pipeline (PGAP) (v2024-07-18.build7555) [33] was used, setting the target organism as *L. clevelandensis*. Functional annotation and gene assignment to Clusters of Orthologous Groups (COGs) were performed using eggNOG-mapper (v2.1.12) [34] with the eggNOG v5.0 database [35]. For the Roary (v.3.13.0) [36] pan-genome analysis, *L. clevelandensis* assemblies and annotations were downloaded from the NCBI Genome database (accessed on 10 April 2025), setting a minimum of 90% completeness and a maximum of 1% contamination threshold calculated on NCBI by the PGAP gene set with the *Actinobacteria* CheckM marker set [37]. The set of used genomes is summarized in Table 1. Core genes were defined as those that are part of a gene family found in more than 90% of the analyzed genomes.

Phylogenetic analysis was performed based on the amino acid sequences of the *transaldolase* gene [38] using the 12 genomes of the 14 available *L. clevelandensis* assembled genomes in the NCBI Genome database. The gene-tree was constructed [39] based on multiple sequence alignment by MAFFT (v7.490) [40]. The best substitution model was selected by functions of phangorn (v2.11.1) package [41] based on the Bayesian information criterion. Bootstrap values were produced by 100 iterations. All data processing and plotting were performed in the R-environment [42].

## 3. Results

The generated raw library size was 160.85 Mb of 104,220 reads, with a mean read length 1543.4, read length N50 3056, and mean read quality 8.0. According to taxon classification, the bacterial genera detected with a relative abundance of at least 1% are listed in descending order: *Porphyromonas* (30.7%), *Bacteroides* (10.9%), *Mobiluncus* (4.1%), *Fusobacterium* (1.7%), *Prevotella* (1.6%), *Corynebacterium* (1.4%), *Ezakiella* (1.4%), and *Peptoniphilus* (1.2%). The *Pasteurella* genus (cultured from the sample by classical approach) was represented by 0.06% of the reads. In total, 8.8% of the reads (n = 7088, length range between 150 and 29,115 bp, with a median of 1626 bp) were identified as *L. clevelandensis*.

The only ARG identified with 100% sequence identity is *EXO-1* (ARG length coverage: 17.20%), an ARG from the EXO beta-lactamase gene family affecting penams through the resistance mechanism of antibiotic inactivation. Based on the VFDB, no virulence factor was detected.

From cleaned reads, 33 fragments were assembled (longest contig: 309,046; mean depth: 9.44). The comparison of the genome to the reference genome ASM129312v1 resulted in an ANI of 89.92%. Based on the CheckM2 report, the MAG reached a 93.49% completeness and 0.42% contamination rate. The assembly had a contig N50 of 92,518 bp and a genome size of approximately 1.91 Mbp (1,909,339 bp). The coding density was 88.5%, with an average gene length of 321 bp, resulting in a total of 1727 genes (1628 excluding pseudogenes/RNAs) and 1682 coding sequences (CDSs) based on the PGAP prediction. Additionally, it features 3 rRNA genes, 48 tRNA genes, and 2 tmRNA genes. The GC content of the MAG was estimated at 59%. No plasmids and phages were identified. (The genome can be accessed on GitHub and is uploaded to NCBI by BioProject PRJNA1045271. The GitHub link is https://github.com/tadrigreta/L_clevelandensis_UVMB (accessed on 25 April 2025)).

**Figure 1:**
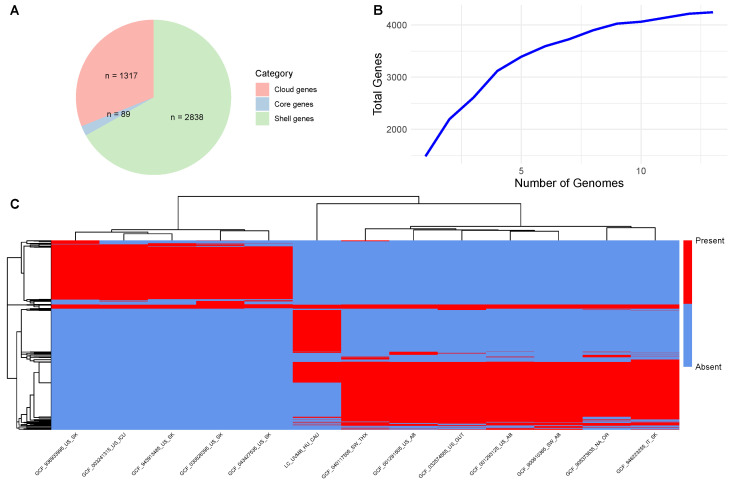
Pan-genome structure of *Lawsonella clevelandensis* strains. (**A**) The number of gene families in each gene set. The categories indicate the proportion of genomes in which a gene family is present: Core genes (90–100%), shell genes (15% to <89%), and cloud genes (0% to <15%). (**B**) Gene accumulation curves for the pan-genome. (**C**) Gene presence–absence heatmap visualizing the distribution of gene families across genomes. Each row corresponds to a gene family, and each column represents an individual strain. Red and blue indicate gene presence and absence, respectively. Both rows and columns are hierarchically clustered based on Euclidean distance.

**Figure 2:**
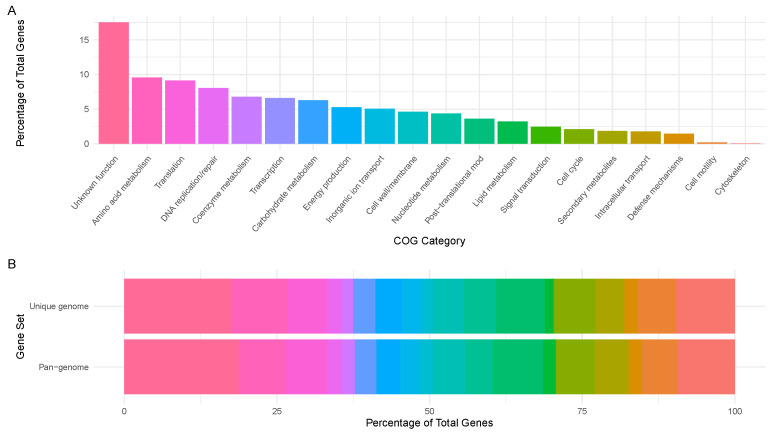
COG functional annotation. (**A**) Percentage rates of COG functional categories associated with the *L. clevelandensis* assembly. (**B**) Comparison of the *L. clevelandensis* pan-genome and the studied genome. The COG categories are represented by the same colors in plots (**A**,**B**).

To explore the genomic composition of L. clevelandensis, genomes from 13 strains were analyzed through pan-genome analysis. The gene accumulation curve indicated an open pan-genome, with the total number of gene families (4244) increasing steadily as more genomes were included. Among these, 89 were identified as core genes (present in ≥90% of genomes), while 2838 were classified as shell genes (found in 15–89% of genomes), and 1317 as cloud genes (present in <15% of genomes). No soft core genes (89% ≤ strains < 90%) were detected in the dataset. Further pan-genome results are summarized in Figure 1. The rates of the Clusters of Orthologous Groups (COGs) extracted from the studied genome and the pan-genome are presented in Figure 2.

Based on the functional annotation, 276 gene families fell to function unknown (S), 151 to amino acid metabolism and transport (E), 144 to translation (J), 127 to replication and repair (L), 107 to coenzyme metabolism (H), 104 to transcription (K), 99 to carbohydrate metabolism and transport (G), 83 to energy production and conversion (C), 80 to inorganic ion transport and metabolism (P), 73 to cell wall/membrane/envelope biogenesis (M), 69 to nucleotide metabolism and transport (F), 57 to post-translational modification, protein turnover, chaperone functions (O), 51 to lipid metabolism (I), 39 to signal transduction (T), 33 to cell cycle control and mitosis (D), 29 to secondary structure (Q), 28 to intracellular trafficking and secretion (U), 23 to defense mechanisms (V), 3 to cell motility (N), and 1 to cytoskeleton (Z). No members of RNA processing and modification (A), chromatin structure and dynamics (B), coenzyme metabolism (H), nuclear structure (Y), or general functional prediction only (R) categories were identified. The functional category rates and the comparison of the functional categories of the studied genome and the pan-genome are presented in Figure 2.

**Figure 3:**
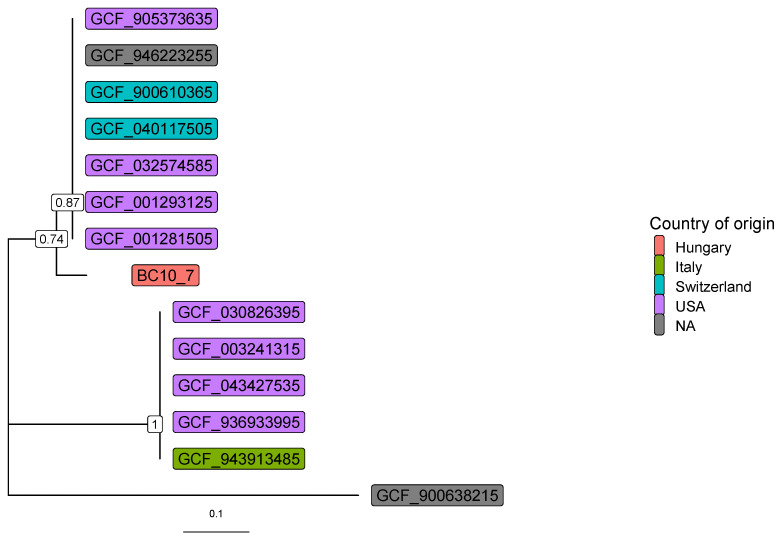
Gene-tree based on *transaldolase* amino acid sequences. RefSeq assembly IDs are shown, and the *Nocardiopsis dassonvillei* (GCF_900638215) was used as an outgroup. Numbers at branches indicate bootstrap support levels (100 replicates). The assemblies originate from abscesses (GCF_001281505, GCF_001293125, GCF_900610365), thorax (GCF_040117505), hospital NICU environment (GCF_003241315), human gut (GCF_032574585), oral cavity (GCF_905373635), and skin metagenomes (GCF_030826395, GCF_043427535, GCF_936933995, GCF_943913485, GCF_946223255).

Figure 3 shows the gene-tree based on the amino acid sequences of the *transaldolase* gene with the best substitution model.

The time taken to complete the steps in the wet lab and dry lab workflows is outlined below. The DNA extraction took around 30 min. The quantification and library preparation steps, including the loading of the flow cell, took around 90 min. The sequencing time was approximately 130 min. The library conversion took 20 s, basecalling 24 s, trimming and filtering 3 min 7 s, taxon classification 1 min 42 s, extraction of *L. clevelandensis* reads 7 s, virulence factor detection 4 s, ORF prediction 1 min 3 s, ARG alignment 37 s, de novo assembly 6 min 24 s, contig polishing 3 min 18 s, scaffolding 1 s, ANI calculation 15 s, and genome annotation 30 min 21 s. The whole process took approximately 300 min, i.e., 5 h. The duration of clinically relevant bioinformatics steps before the assembly was about 4.3 h.

## 4. Discussion

*L. clevelandensis* has been detected in multiple human sources associated with health issues, including dermatitis [43,44], various abscesses [44,45,46,47,48,49], gut dysbiosis [50], and vascular graft infection [6,7,51]. Other studies showed that the bacterium can be found in the microbiome of skin [52], nostrils [53], human hair follicles [54], public transit air [55], or even worn spectacles [56]. Further investigations on the human skin microbiota have revealed interesting findings related to *Lawsonella* spp. Within a next-generation sequencing-based study, negative correlations among *L. clevelandensis* and the pathological state of human atopic dermatitis were found [57]. Throughout the work of another research group, *Lawsonella* quantities were found to have a positive correlation with transdermal water loss and a negative correlation with the skin water content. This finding indicates the emergence of this bacterium when the skin’s integrity is impaired [58].

To our knowledge, just a few studies have so far shown the presence of *L. clevelandensis* in animals. Within a study, the genome fragments of a member of the *Lawsonella* genus were identified in bull semen by 16S rRNA sequencing [59]. In a further study, *L. clevelandensis* was detected in the oral microbiome of primates using shotgun metagenomics [60]. Furthermore, in a 16S rRNA sequencing study assessing the mucosal microbiome in equine glandular gastric disease, the *Lawsonella* genus was found to be associated with the healthy state of the microbiome rather than the pathological conditions [61].

Besides our assembly, the NCBI Genome database (accessed on 10 April 2025) has 14 *L. clevelandensis* assemblies, all originating from human or human environmental sources. Furthermore, to date, publications including genome sequences of *L. clevelandensis* have also been assembled from human isolates [62,63]. As a complement to this series of human isolated *L. clevelandensis* genomes, the presented assembled genome derives from an animal.

The comparisons of the newly described animal-derived genome and the genomes of human origin provided substantial knowledge of the species. The relatively small set of core genes indicates considerable genetic diversity among the strains (Figure 1). Moreover, the high proportion of cloud genes (1317 out of 4244) underscores the species’ heterogeneity, suggesting a pronounced level of genome plasticity. As displayed on Figure 1C, the gene presence–absence heatmap revealed three distinct clusters of gene families, each associated with specific subsets of the 13 analyzed strains. Two major clusters were linked to groups of seven and five strains, respectively, likely reflecting lineage-specific core genes shared within each group and suggesting phylogenetic divergence or ecological specialization. Interestingly, the third cluster representing the animal-derived target strain was exclusive and displayed a unique gene content profile not shared with other genomes. Based on this, the animal-derived strain is an outlier with potentially specific adaptations.

To better understand the functional characteristics of *L. clevelandensis*, the identified genome and the pan-genome were also annotated using the eggNOG database [35]. Furthermore, the functional categories of the pan-genome were compared to the animal-derived study target genome. The relative distribution of the functional categories was similar in the pan-genome reference and the target strain (Figure 2B). A substantial portion of the genome of the strain, based on the pan-genome annotation, was classified as “function unknown (S)”, reflecting genes that encode proteins with unknown functions or lacking homologs beyond the *L. clevelandensis* species. This finding could indicate that specialized adaptations of the species are not well characterized in current databases and can possible indicate the presence of novel biochemical pathways. Besides these findings, a broad range of metabolic capabilities and essential core cellular processes were dominating besides moderate defense mechanisms. Very few genes being related to cell motility and the cytoskeleton is associated with the fact that *L. clevelandensis* is non-motile. No chromatin or nuclear-related processes confirm that the functional annotation was performed on a prokaryote.

As demonstrated in Figure 3, sequence identity among *L. clevelandensis* presented in this study, and other strains of the bacterium, cannot be explained by sample origins or geographic locations. However, the same pattern of seven, five, and one strain composing three clusters can be observed as in Figure 1C.

Importantly, despite the bacterial genome assembly being performed on a sample derived from an inflammatory environment, the role of the bacterium in external otitis cannot be confirmed. Further studies are required to elucidate its exact contribution to the development and maintenance of the disease. However, a possible role in the pathogenesis of external otitis caused by allergodermatitis may partially be explained by its association with the impairment of the skin barrier function. Further studies are warranted to reveal whether *L. clevelandensis* is a transient microbiota component in dogs, or is of human origin. However, if *L. clevelandensis* is considered as the inhabitant of the dog epidermis, its associations with similar skin changes as in the case of humans would be required to be studied. Importantly, in a recent study [64], where the normal microbiota of the external ear canal and middle ear of healthy dogs was examined using 16S rRNA sequencing, *L. clevelandensis* was not found among the constituents of the ear microbiota.

While no antimicrobial resistance (AMR) determinants were identified in the genome of *L. clevelandensis* with low to very low Minimal Inhibitory Concentration (MIC) antimicrobial susceptibility results in previous studies [63], we have identified one ARG hit with lower length coverage but high base sequence identity. Nevertheless, considering the fact that the low coverage of the ARG might potentially lead to a false positive hit or a misannotation, and no gene expression studies or phenotypic AMR testing were performed, the functional significance of this ARG is unknown. Furthermore, consistent with our findings, Goldenberger and colleagues also identified no virulence genes by the Whole Genome Sequencing (WGS) of *L. clevelandensis* [63]. Considering the limited genomic data currently available for *L. clevelandensis*, with only a few genomes deposited in NCBI, the repertoire of species-specific virulence genes potentially harbored by this organism remains largely unknown. Although phenotypic evidence supports its pathogenic potential, the underlying genomic determinants have yet to be comprehensively characterized. Although our understanding of the genotypic characteristics of L. clevelandensis continues to expand, further investigations are essential to elucidate its precise role and underlying mechanisms of pathogenicity.

*L. clevelandensis* is a fastidious, anaerobic bacterium with a lengthy incubation time of at least 10 days [46]. As such, conventional culture techniques often fail to isolate the bacterium [7,44,51]. Once successfully cultured, the differentiation of *L. clevelandensis* from non-tuberculous mycobacteria is still demanding due to its partial acid resistance [45]. Therefore, even if diagnostic microbiology laboratories target the pathogen, culturing and identification during the routine bacteriological testing period are unlikely. Nanopore sequencing and consecutive bioinformatic data processing enable the detection of the microorganisms, combined with assessing their abundance rates, virulence genes, and antimicrobial resistance determinants within 4–24 h [65]. The sample processing time of approximately 5–6 h required for both wet and dry lab processes is in line with the study by Ring and colleagues, who also found 4–8 h to be sufficient to obtain full results from one sample [65]. Concurrently, our calculations indicate that attaining an adequate sequencing depth with a MinION sequencer and the specified sequencing conditions for a single metagenomic sample would require 130 min using a flow cell with available nanopore numbers above the warranty level (800 pores) at the start. Nevertheless, the most optimal scenario, as depicted by Oxford Nanopore Technologies (https://nanoporetech.com/products/sequence/minion, accessed on 26 November 2024), suggests that even 13 min could be sufficient to achieve the same sequencing depth using a MinION with a MinION R10.4.1 flow cell with a sequencing speed of 400 bases/second/pore and 512 active nanopores. However, sequencing time could be significantly reduced using PromethION sequencers and PromethION R.10.4.1 flow cells. By this platform, the most optimal scenario for a sample of the same sequencing depth would be 2.5 min with a sequencing speed of 400 bases/second/pore and 2675 active nanopores (https://nanoporetech.com/products/sequence/promethion, accessed on 26 November 2024). In parallel with the MinION results, it can be reasonably deduced that the generation of an optimal data volume for the specified metagenomic purpose would realistically require approximately 10 min when utilizing the PromethION platform with a flow cell that has a minimum initial availability above the warranty level (5000 nanopores). Thus, ONT-based clinical metagenomics may become an important tool for diagnostic microbiology and clinical work. In agreement with recent next-generation sequencing (NGS)-based studies, we find this approach particularly important in the complementary diagnosis of infections with an unsuspected cause that are potentially caused by *L. clevelandensis*, especially when conventional diagnostic methods are inconclusive and too slow [44,66,67]. In the case study of Lijia and colleagues, the effective antibiotic treatment for recurrent skin infections and multiple abscesses was successfully selected after the sequencing-based identification of *L. clevelandensis* [44]. In their publication of a series of osteoarticular infection cases, Petri and colleagues also present the detection of *L. clevelandensis*, an unsuspected diagnosis by nonvertebral osteomyelitis [66]. Furthermore, in line with Watanabe and colleagues, we found that even non-invasive swabbing is sufficient to collect genomic material for detecting *L. clevelandensis* and there is no need for more intrusive sampling techniques [68]. Furthermore, as demonstrated on our target strain, the ability to quickly detect mobile genetic elements, such as plasmids or phages, contributes to the favorable public health potential.

Nevertheless, the novelty of this approach leaves many questions unanswered, such as discrepancies between the detected antimicrobial resistance genes and the phenotypic antimicrobial resistance expected [69]. Similarly, the pathogenic role of microorganisms identified solely by metagenomics in diseases previously associated with one or a few different pathogens needs to be comprehended [70]. Similarly, pathogenetic correlations of the abundance of detected microorganisms may also open new perspectives in learning, better treating, and hindering the development of diseases with an infectious background in the future.

It should also be noted that this often overlooked, opportunistic pathogenic bacterium, which has only recently been described phenotypically and genotypically [5,62], was identified in the ear canal of a companion animal. At the same time, a change in the quality of human–pet bonds is notable. The current mindset often enhances physical proximity (e.g., sleeping with the owner, unhygienic interactions causing human exposure to canine saliva and other secretions, etc.) among dogs and their owners. Specifically, around 70% of owners regard and treat their dogs as family members, 17% as companions, and only 3% as property, according to a survey by the American Veterinary Medical Association [71]. Considering the nature of modern human–pet bonds [72], the possibility is raised that this emerging pathogenic bacterium may have zoonotic significance and, thus, enhanced One Health implications. The growing body of knowledge on this often-neglected pathogen can contribute to developing better treatment options and preventive measures. Furthermore, the results promote the use of rapid, non-targeted shotgun metagenomic sequencing performed with nanopore sequencers that can grant the identification of microorganisms that are difficult to culture.

## Figures and Tables

**Table 1:** Metadata of used *L. clevelandensis* genomes. All of the available genomes are of human origin.

NCBI RefSeq	BioProject	Collection	Country	Origin
**ID**	**ID**	**Year**		
GCF_001281505	PRJNA256353	2013	USA	Peritoneal abscess
GCF_001293125	PRJNA256353	2011	USA	Abscess
GCF_003241315	PRJNA376580	2014	USA	NICU environment
GCF_030826395	PRJNA872116	2019	USA	Skin
GCF_032574585	PRJNA294605	2014	USA	Infant ICU gut
GCF_040117505	PRJNA1095233	2023	Switzerland	Lung abscess
GCF_043427535	PRJNA987158	2021	USA	Swine farm worker skin
GCF_900610365	PRJEB29478	2018	Switzerland	Breast abscess
GCF_905373635	PRJEB43277	2021	Unknown	Oral cavity
GCF_936933995	PRJEB51076	2023	USA	Skin
GCF_943913485	PRJEB47281	2022	USA	Skin
GCF_946223255	PRJEB47281	2022	Italy	Skin

## Data Availability

The raw long-read data (SRR26949354) of the sample (SAMN38440151) are publicly available and accessible through the PRJNA1045271 from the NCBI Sequence Read Archive (SRA).
